# Potts Shunt to Be Preferred Above Atrial Septostomy in Pediatric Pulmonary Arterial Hypertension Patients: A Modeling Study

**DOI:** 10.3389/fphys.2018.01252

**Published:** 2018-09-10

**Authors:** Tammo Delhaas, Yvette Koeken, Heiner Latus, Christian Apitz, Dietmar Schranz

**Affiliations:** ^1^Department of Biomedical Engineering, CARIM School for Cardiovascular Diseases, Maastricht University, Maastricht, Netherlands; ^2^Department of Paediatric Cardiology and Congenital Heart Defects, German Heart Centre, Munich, Germany; ^3^Division of Pediatric Cardiology, University Children’s Hospital Ulm, Ulm, Germany; ^4^Pediatric Heart Center, Justus Liebig University Giessen, Giessen, Germany

**Keywords:** computer modeling, pulmonary arterial hypertension, Potts shunt, atrial septostomy, exercise, pump work, shunt flow, oxygen saturation

## Abstract

**Aims:** To quantitatively evaluate the basic pathophysiological process involved in the creation of Eisenmenger syndrome in pediatric pulmonary arterial hypertension (PAH) patients by either atrial septostomy (AS) or Potts shunt (PS) as well as to predict the effects of AS or PS in future PAH patients.

**Methods:** The multi-scale lumped parameter CircAdapt model of the cardiovascular system was used to investigate the effects of AS and PS on cardiovascular hemodynamics and mechanics, as well as on oxygen saturation in moderate to severe PAH. The reference simulation, with cardiac output set to 2.1 l/min and mean systemic pressure to 61 mmHg, was used to create a compensated moderate PAH simulation with mPAP 50 mmHg. Thereupon we created a range of decompensated PAH simulations in which mPAP was stepwise increased from 50 to 80 mmHg. Then we simulated for each level of mPAP the acute effects of either PS or AS with connection diameters ranging between 0–16 mm.

**Results:** For any mPAP level, the effect on shunt flow size is much larger for the PS than for AS. Whereas right ventricular pump work in PS is mainly dependent on mPAP, in AS it depends on both mPAP and the size of the defect. The effects on total cardiac pump work were similar for PS and AS. As expected, PS resulted in a drastic decrease of lower body oxygen saturation, whereas in AS both the upper and lower body oxygen saturation decreased, though not as drastically as in PS.

**Conclusion:** Our simulations support the opinion that a PS can transfer suprasystemic PAH to an Eisenmenger physiology associated with a right-to-left shunt at the arterial level. Contrary to the current opinion that PS in PAH will decompress and unload the right ventricle, we show that while a PS does lead to a decrease in mPAP toward mean systemic arterial pressure, it does not unload the right ventricle because it mainly diverts flow from the pulmonary arterial system toward the lower body systemic arteries.

## Introduction

Idiopathic pulmonary arterial hypertension (iPAH) is a rare, progressive, and ultimately devastating disease if left untreated. The disease will lead to a gradual increase in right ventricular (RV) pressure, right heart failure, functional incapacity, and death. Though recent advances in medical treatment have improved survival and quality of life in both adult and children, the mortality rate is still high. Currently freedom from death or transplantation in pediatric iPAH patients is less than 60% at 5 years (Moledina et al., 2010), highlighting the need for novel therapeutic approaches.

Life expectancy of patients with Eisenmenger syndrome, representing the end stage of an untreated left-to-right shunt in which pulmonary arterial hypertension (PAH) prohibits intracardiac repair, is significantly superior to that of iPAH patients. The median survival in Eisenmenger syndrome is estimated between 40 and 60 years, depending on the underlying cardiac defect (Hoffman, 2009). Converting cardiovascular features of iPAH patients to features typical of Eisenmenger syndrome by means of atrial septostomy (AS) is therefore an additional strategy in both adult and pediatric iPAH patients with recurrent syncope or significant right heart failure refractory to medical treatment (Barst, 2000; Micheletti et al., 2006). The created atrial septal defect (ASD) permits a right-to-left shunting that results in RV unloading and maintenance or increase of systemic flow despite severe pulmonary disease. Though AS improves symptoms and quality of life in adults and children with severe iPAH, the sizing of the defect is critical since too much right-to-left shunt at the atrial level could be immediately life threatening because of insufficient pulmonary blood flow as well as severe desaturation in the brain and in the coronary circulation, and too less shunting may require repeated procedures because of spontaneous closure of the defect (Micheletti et al., 2006). Another approach to convert iPAH patients to Eisenmenger physiology arose a decade ago and calls for the creation of an anastomosis between the descending aorta and the left pulmonary artery (Blanc et al., 2004). This so-called Potts shunt (PS) has the advantages that it does not provoke oxygen desaturation in the upper part of the body and that it directly limits pulmonary pressures to isosystemic values. The sparse literature on surgical creation of PS in pediatric iPAH patients report a success rate in the order of 75% (Blanc et al., 2004; Baruteau et al., 2012, 2015; Petersen et al., 2013). Transcatheter creation of PS by stenting a patent ductus arteriosus (PDA) in pediatric iPAH patients had a comparable clinical success rate outcome (Boudjemline et al., 2013; Esch et al., 2013; Latus et al., 2014; Boudjemline et al., 2017).

The very sparse literature on the creation of Eisenmenger syndrome in pediatric iPAH patients by either AS or PS does not quantitatively evaluate the basic pathophysiological process involved, nor can it be used to predict the effects of AS or PS in future iPAH patients. We therefore applied CircAdapt, our lumped-parameter mathematical model of the heart and circulation, to quantitatively evaluate the acute hemodynamic effects of AS and PS in pulmonary hypertension. Simulations were performed for shunt sizes with diameters ranging from 0 to 16 mm, while using the average patient as reported by Baruteau et al. (2012) as reference, albeit that mean pulmonary arterial pressures (mPAPs) ranged from 3/4 to 4/3 mean systemic arterial pressure.

## Materials and Methods

### Lumped Parameter Modeling Approach

We applied the multi-scale lumped parameter CircAdapt model of the cardiovascular system to investigate the effects of AS and PS on cardiovascular hemodynamics and mechanics, as well as on oxygen saturation in pulmonary hypertension with mPAPs ranging from 3/4 to 4/3 mean systemic arterial pressure. The CircAdapt hemodynamic model of the heart and circulation (Arts et al., 2005; Lumens et al., 2009; Koeken et al., 2012; Walmsley et al., 2015) allows simulation of beat-to-beat mechanics and hemodynamics of the human cardiovascular system for both research and educational purposes^1^. CircAdapt is built on physiological and physical principles, described in brief below; more detailed theoretical descriptions of the CircAdapt model have been published previously (Arts et al., 2005, 2011, 2012; Lumens et al., 2009; Koeken et al., 2012; Walmsley et al., 2015). The model consists of modules for myofibre stress and strain, atrial and ventricular pressure, flow across cardiac valves, blood pressure within major veins and arteries, and systemic and pulmonary resistances. The right and left ventricles are mechanically coupled through the inter-ventricular septum (Lumens et al., 2009). Mean aortic pressure and cardiac output can be controlled through changes to systemic peripheral resistance and total blood volume, mimicking homeostatic control mechanisms. CircAdapt uses physiological structural remodeling rules in response to mechano-sensed signals to produce a realistic geometry of the heart and circulation (Arts et al., 2012). The dynamic behavior of the CircAdapt model allows blood to move in both directions through all cavities, shunts, and vessels during the complete cardiac cycle. Mixing of flows with different oxygen concentrations and saturations is described by differential equations that take in- and outlet blood flow sizes and oxygen concentrations into account (Koeken et al., 2012). Oxygen uptake in the lungs is assumed to render an oxygen saturation of 98% in the pulmonary veins, whereas oxygen consumption for the body as a whole can be prescribed and for the various organs can be made dependent on their size and/or work.

Modeling principles key to this simulation study include that time-depending pulmonary circulating blood flow is defined a function of a reference flow and a (normalized) pulmonary pressure drop:

$$\begin{array}{c} q_{pulm}\left(t\right)=\frac{q_{pulm,ref}}{\Delta p_{pulm,ref}}\Delta p_{pulm}(t), \end{array}$$

with q_pulm,ref_ and q_pulm_ the (reference) pulmonary circulating flow and Δp_pulm,ref_, and Δp_pulm_ the (reference) pulmonary pressure drop. Based on this relation, mPAP can be modulated by adjusting the reference pulmonary pressure drop (Δp_pulm,ref_). To create PAH with mPAPs ranging from 50 to 80 mmHG, Δp_pulm,ref_ was varied between 44.3 and 77.5 mmHg.

Furthermore, the CircAdapt’s cardiac valve module is utilized for creation of an ASD or PS. Briefly, for an ASD a valve element is incorporated connecting the left- and right atria, whereas for a PS, a valve element is incorporated connecting the pulmonary arteries to the descending aorta. In contrast to cardiac valves, however, the valves forming either a ASD or an PS, are characterized by having a constant diameter over time.

### Simulation Protocol

Specific input parameters for our modeling study were derived from data presented in the paper by Baruteau et al. (2012) on the short and long term effects of a PS in eight children diagnosed with iPAH. For the reference simulation we set systemic blood flow (2.1 l/min) and mean arterial pressure (61 mmHg) to the average values of the iPAH children studied, whereas mPAP was set to 15 mmHg. Thereafter, cardiac wall sizes and blood vessel diameters were adapted to the mechanical load of the tissues during exercise with a threefold increase in cardiac output and a twofold increase in heart rate. Thereafter we simulated moderate PAH with an mPAP of 50 mmHg in rest with adaptation of heart and vessels to exercise with a twofold increase in cardiac output and a 1.8-fold increase in heart rate. Using this compensated moderate PAH simulation as a starting point, and keeping cardiac output at 2.1 l/min, we created two matrices of simulations. First we created a range of decompensated PAH simulations by stepwise increasing pulmonary resistance in absence of structural cardiovascular adaptation. This resulted in a range of simulations with 5 mmHg increases in mPAP from 50 till 80 mmHg, the mPAP-level of the average “Baruteau-child.” Then we simulated for each level of mPAP the acute effects of either an ASD or a PS which diameter ranged between 0–16 mm. Our simulation protocol finally yielded two sets of 119 (=7 × 17) simulations.

## Results

**FIGURE 1:**
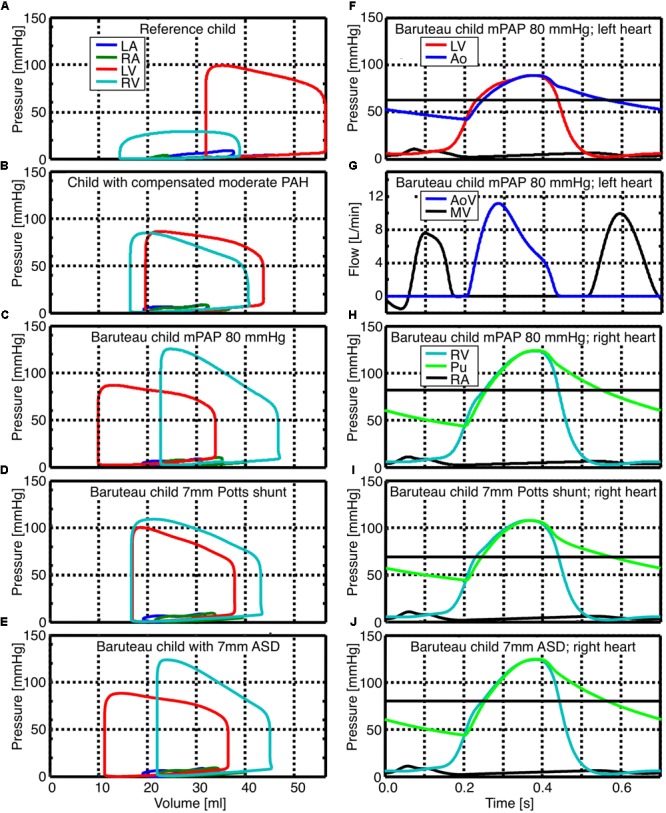
Simulated pressure-volume loops as well as pressure and flow curves for reference child (panel **A**), child with compensated moderated pulmonary hypertension (panel **B**), the average “Baruteau child” with suprasystemic mean pulmonary artery pressure of 80 mmHg (panels **C, F, G**, and **H**), as well as the “Baruteau-child” with either a 7 mm Potts shunt (panels **D** and **I**) or a 7 mm ASD (panels **E** and **J**). Horizontal solid black line in panels with pressure curves indicates the mean systemic (panel **F**) or mean pulmonary arterial pressure (panels **H, I**, and **J**). Abbreviations: Ao, aorta; AoV, aortic valve; LA, left atrium; LV, left ventricle; mPAP, mean pulmonary arterial pressure; PAH, pulmonary arterial hypertension; Pu, Pulmonary artery; RA, right atrium; RV, right ventricle.

Pressure-volume relations (pV-loops) for the left atrium (LA) and ventricle (LV) as well as the right atrium (RA) and ventricle (RV) are shown in the left panels of **Figure 1** for the reference child (**Figure 1A**), child with compensated moderate PAH (**Figure 1B**), the average “Baruteau-child” with decompensated PAH and a suprasystemic mPAP of 80 mmHg (**Figure 1C**), as well as for the average “Baruteau-child” with either a 7 mm PS (**Figure 1D**) or a 7 mm ASD (**Figure 1E**). With increasing mPAP, RV pV-loops shifted to the right, most prominently in the “Baruteau-child” with decompensated PAH. The concomitant interventricular septal bowing to the left caused a decrease in left ventricular (LV) volumes, leading to a leftward shift of the LV pV-loops (**Figures 1A–C**). After creation of a 7 mm PS (**Figure 1D**), the RV pV-loop shows an increased stroke volume but lower RV systolic pressure. This results in approximately the same pV-loop area as before the PS, indicating that RV workload is hardly affected. The LV pV-loop shows an increased systolic pressure and a slightly smaller stroke volume, albeit that the loop is shifted to the right. Also LV pV-loop area is approximately the same as before the PS. Creation of a 7 mm ASD shunt (**Figure 1E**) resulted in a right-left shunt at atrial level with concomitantly decreased RV and increased LV stroke volume, whereas systolic pressures were hardly affected at both sides. Consequently, RV workload decreased slightly whereas LV workload increased. The right panels of **Figure 1** show time curves of pressures and flows for the average “Baruteau-child” with mPAP of 80 mmHg (**Figures 1F–H**) as well as of right heart pressures for the “Baruteau-child” with either a 7 mm PS (**Figure 1I**) or a 7 mm ASD (**Figure 1J**). The latter panels clearly show that whereas a PS results in lowering of mPAP toward mean systemic arterial pressure, an ASD barely influences right heart pressures.

**FIGURE 2:**
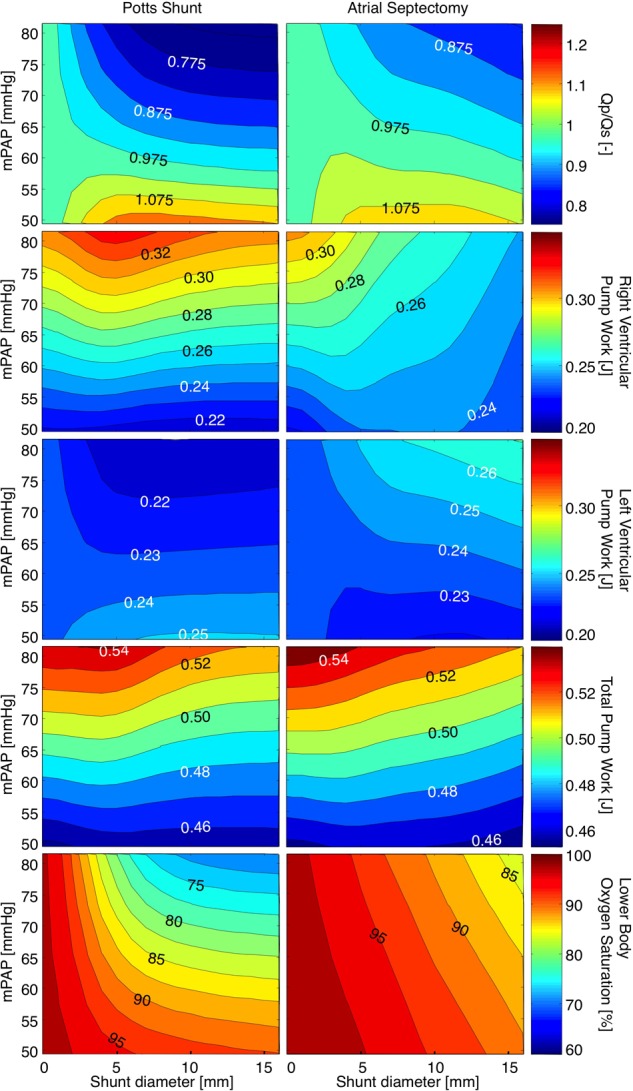
Heatmaps for the ratio of pulmonary to systemic flow (Qp/Qs), right ventricular-, left ventricular-, and total pump work, as well as lower body oxygen saturation under various levels of mean pulmonary arterial pressure (mPAP) with shunt diameters ranging from 0 to 16 mm. The left panels show the immediate effects of Potts shunts, while the results for atrial septostomy are displayed in the right panels.

**Figure 2** shows heatmaps for the ratio of pulmonary to systemic flow (Qp/Qs), RV-, LV-, and total pump work, as well as lower body oxygen saturation under various levels of mPAP before shunt creation and with shunt sizes ranging from 0 to 16 mm. The left panels show the immediate effect of PSs, while the results for AS are displayed in the right panels. For PS sizes up to 7 mm, the size and the direction of the shunt flow depend both on mPAP and shunt size. For shunt diameters over 7 mm, Qp/Qs is only proportional to the extent of pulmonary hypertension. AS shows qualitatively the same relation between defect size, mPAP and Qp/Qs, though changes in Qp/Qs are less. In the absence of shunts, RV pump work increased with increasing mPAP. The effects of shunt size on RV pump work are not similar for PSs and AS. Whereas RV pump work was barely influenced by PS sizes (only PSs with diameters of 4 to 5 mm resulted in a small increase in RV pump work), increasing AS defect sizes clearly resulted in a proportional decrease in RV pump work. LV pump work was barely influenced by small AS defects and small PS sizes. As of shunt sizes of 3 mm, PS and AS had opposite effects on LV pump work. In the presence of PS over 3 mm diameter in size, LV pump work decreased with suprasystemic mPAP and increased when mPAP was below mean systemic pressure. In the presence of AS defect sizes over 3 mm diameter, LV pump work increased with suprasystemic mPAP and decreased when mPAP was below mean systemic pressure. Heat maps for total pump work showed that mPAP strongly determines total pump work. For each level of mPAP, PSs up to 5 mm diameter barely influenced total pump work, whereas total pump work increased with roughly 2% when PS sizes increased from 5 to 16 mm diameter. AS defect sizes were inversely related to total pump work at each level of mPAP, with total pump work on average 2% lower when AS defect size increased to 16 mm. Whereas PS did not influence upper body arterial oxygen saturation (results not shown), it induced a decrease in lower body arterial oxygen saturation, especially for high mPAP and small shunt diameters. AS led to a decrease in both upper and lower body arterial oxygen saturation that was proportional to both mPAP and defect size.

## Discussion

The CircAdapt model of the human heart and circulation has been used to evaluate the effect of PS and AS on both hemodynamics and oxygen saturation for the average PAH patient that underwent a PS in the original report by Baruteau et al. (2012). We show that for any mPAP level, the effect on shunt flow size is much larger for the PS than for the created ASD. Whereas RV pump work in PS is mainly dependent on mPAP, in AS it depends on both mPAP and the size of the created ASD. Though the effect on total cardiac pump work were similar for PS and AS, the effect on upper and lower body oxygen saturation were not. In PS, upper body oxygen saturations were not effected and remained at pulmonary venous levels, whereas lower body oxygen saturation drastically decreased. In AS, the effect of shunt size on decrease of oxygen saturation were similar for the upper and lower body, though not as drastically as in PS.

Because the size of the PS is thought to be critical for the extent of right-to-left or left-to-right shunt, measures have been taken in clinical practice to limit the size of the created connection. Surgically created PS sizes are aimed for to be in the order of the diameter of the descending aorta (Baruteau et al., 2012, 2015), which is approximately 10 mm for children with a body surface area of 0.75 m^2^ (Kaiser et al., 2008). If the PS is created by stenting a PDA, the diameter of the ductal stent connection is gradually increased by sequential balloon inflations with the aim of equalizing the PA and aortic systolic pressures, maintaining pulmonary blood flow and limiting the desaturation of the inferior limbs (Baruteau et al., 2015). In our simulations, however, we didn’t see hardly any change in Qp/Qs above PS diameters of 7 mm. From 0 to 7 mm, shunt flow was positively related to the shunt diameter as well as to the difference between mPAP and sPAP. Therefore it seems safe to aim immediately for a PS size in the order of the descending aorta diameter, be it surgically created or by means of stenting the PDA.

In patients with (infra)systemic level of pulmonary artery pressures, creation of a Potts anastomosis bears the risk of turning the interarterial right-to-left shunt into a left-to-right shunt, that on it’s turn presumably can lead to a volume overload of the pulmonary circulation, increased pulmonary artery pressures and a steal phenomenon from systemic to pulmonary circulations (Baruteau et al., 2015). We show that with infrasystemic mPAP, Qp/Qs rises in the acute situation, but that neither the RV nor the LV have additional load. However, because Qp rises on the expense of Qs, patients with infrasystemic PAH bears the risk of developing syncope upon creation of a PS. We therefore advocate the use of unidirectional valved Potts anastomosis, of which promising results have been shown in an animal model of PPH (Bui et al., 2011) as well as in the first human case ever (Baruteau et al., 2015).

In our simulations, the diversion of pump work from the RV to the LV was more pronounced in AS than in PS. However, since AS also results in coronary artery oxygen desaturation, the cardiac exercise capacity of PS will be well above the one in AS. In PAH patients with PS, the needed increases in systemic flow with exercise will not be on the expense of diminished oxygen transport to the myocardium. In a follow-up study on 24 PAH patients with PS, none of them had syncope or right heart failure (Baruteau et al., 2015). Hence, a permanent post-cardiac right-to-left shunt is to be preferred above an intra-cardiac one.

Modeling studies can provide valuable mechanistic insights in cardiovascular function dynamics before and after PS or AS in PAH patients. In a previous modeling study we provided evidence that AS might only be beneficial to patients with severe PH and that this benefit originates from an increase of LV preload and not from an increase in oxygen delivery to the tissue, as stated in previous studies (Kerstein et al., 1995; Sandoval et al., 1998; Rothman et al., 1999; Micheletti et al., 2006; Kurzyna et al., 2007; Galie et al., 2009; Zierer et al., 2009). In the current study, our simulations indicate that a PS in PAH will lead to a decrease in mPAP toward mean systemic arterial pressure, but that the PS does not unload the RV because it mainly diverts flow from the pulmonary arterial system toward the lower body systemic arteries. Whereas the current study assessed the effects of shunt type and size in an average child with PAH, we foresee an inverse modeling approach, in which the model is personalized by fitting simulated hemodynamics to a set of patient-specific data, to predict outcome of intended interventions, albeit that detailed cardiac morphology and flow dynamics, which could exert significant influences on the outcome, are not taken into consideration in our modeling approach.

## Conclusion

Our simulations support the opinion that a PS can transfer suprasystemic PAH to an Eisenmenger physiology associated with a right-to-left shunt at the arterial level. Contrary to the current opinion that PS in PAH will decompress and unload the right ventricle, we show that while a PS does lead to a decrease in mPAP toward mean systemic arterial pressure, it does not unload the right ventricle because it mainly diverts flow from the pulmonary arterial system toward the lower body systemic arteries.

## Data Availability Statement

The simulation software used for this study can be found at www.circadapt.org.

## Author Contributions

TD, CA, and DS conceived and designed the study. TD and YK performed the simulations and created the figures. TD wrote the first draft of the manuscript. All authors contributed to manuscript revision, read, and approved the submitted version.

## Conflict of Interest Statement

The authors declare that the research was conducted in the absence of any commercial or financial relationships that could be construed as a potential conflict of interest.

## Footnotes

1 www.circadapt.org
